# Participant Engagement, Epistemic Injustice, and Early‐Phase Implanted Neural Device Research

**DOI:** 10.1002/hast.70022

**Published:** 2025-10-15

**Authors:** Lilyana Levy, Ashley Feinsinger

**Keywords:** neuroethics, disability, participant engagement, brain‐computer interfaces, neurotechnology, epistemic injustice, bioethics

## Abstract

*In recent years, participant engagement initiatives in research on implanted neural devices have significantly increased. However, there remains little consensus on the motivations, goals, and best practices for engagement efforts. Drawing on the concept of* participatory epistemic injustice, *we argue that one core ethical motivation for engagement is epistemic in nature. Based on their subject positions, participants should be key knowledge contributors to implanted neurotech research. Therefore, we argue, participants experience participatory epistemic injustice when their insights do not result in changes to or otherwise influence research protocols, device development, and task design. We contend that engagement can resist this type of injustice only if it establishes robust methods not only to gather but also to actively incorporate participant knowledge into the research and development process*.

## Neuroscience & Society

The term “engagement” has become a buzzword in neural device research. Several new initiatives have been launched within the past two years to engage various stakeholders in neural device research and its clinical translation. Engagement efforts have been sponsored by a wide range of entities, including regulatory bodies (for example, the U.S. Food and Drug Administration), funding agencies (such as the National Institutes of Health), neurotechnology companies (including Synchron and Motif Neuro), conferences (such as those of the Brain‐Computer Interface Society), and engineering organizations (including the Institute of Electrical and Electronic Engineers). The efforts incorporate a variety of methods, including collaborative communities, patient advisory boards, engagement workshops, qualitative research with patients and participants, and other codesign methodologies. These efforts are welcome, as engagement is widely embraced in many contexts as the mark of good clinical research and ethical treatment of research participants.

However, there is a great deal of inconsistency across these efforts and, we would argue, confusion along several dimensions about the practice and purpose of engagement in neural device research. Which conceptions of engagement are being invoked in neural device research contexts? What engagement practices are in use, and who is being engaged by these practices? And importantly, why should we embrace engagement in this research, and why should we prioritize engagement with certain people or groups over others?

Uncertainty surrounding these questions may result in simply asking participants to do more labor without meaningfully impacting device research and development. As the Patient‐Centered Outcomes Research Institute stresses, lack of clarity about why it is important to seek participant perspectives is the biggest challenge to engagement efforts.^1^

Gaining clarity about the ethical motivations for engagement in neural device research could lead to answers to these questions. In this paper, we narrow the discussion in two ways. First, we focus on engagement with current or past research participants, as opposed to community engagement, and second, we focus on one underexplored ethical motivation for engagement that may guide its practical implementation in implanted neural device research: epistemic injustice. In what follows, our goal is not to indict, dismiss, or ignore the existing efforts that help elevate participant knowledge in this research. Instead, we aim to clarify why epistemic injustice should be taken seriously as a motivation for engagement in these studies and, secondarily, to suggest how this motivation may reshape the types of engagement that are pursued. Ultimately, we argue, this motivation justifies community‐based participatory research and focused attention on engagement with participants enrolled in these studies.

Drawing on recent philosophical literature about epistemic injustice in health care, we argue that one of the primary ethical motivations for participant engagement in implanted neural device research ought to be epistemic in nature. Leveraging a framework by Ian James Kidd and Havi Carel, we propose that participants in clinical trials of implanted neural devices are subject to *participatory epistemic injustice* when their knowledge does not trigger action regarding or otherwise influence design processes, success parameters, and other clinical research practices. We propose that engagement efforts will be most successful when they recognize participants as key epistemic contributors and incorporate their knowledge into neural device research and development. We describe how engagement driven by concerns for epistemic injustice extends beyond standard neuroethical approaches to conducting qualitative research about participant experiences, values, and preferences. Although these methods allow ethicists to gain nuanced understandings of participant experiences, they do not necessarily involve feedback mechanisms whereby participant knowledge can directly influence future study and device design. Reframing discussions of engagement through the lens of epistemic injustice helps illuminate how and why it is imperative that engagement results in impactful changes to research and development, especially given that the number of people in implanted neural device research is projected to grow.

We start with a brief overview and critique of current engagement practices and their motivations in implanted neural device research. We then introduce the epistemic injustice framework, focusing on Kidd and Carel's analysis of participatory epistemic injustice. Next, we identify three core areas of participant knowledge that are especially valuable for implanted neural device research and are subject to marginalization. Finally, we conclude with some preliminary suggestions for how these core areas of participant knowledge may guide engagement efforts in implanted neural device studies, focusing on the value of engaging enrolled participants alongside other community efforts. 

## Engagement: The State of the Field

Engaging with relevant groups of people—whether they are patients, advocacy groups, the public, or participants in studies—is increasingly recognized as a core feature of good clinical research. In its broadest sense, the concept of participant engagement in clinical research, including with current and past research participants, encompasses a cluster of approaches that aim to involve participants beyond their traditional, passive roles as research subjects. It has been described by the Food and Drug Administration (FDA) as, “intentional, meaningful interactions with patients that provide opportunities for mutual learning, and effective collaborations.”^2^ Engagement with participants in clinical studies can embody a variety of activities, including soliciting feedback, seeking consultation, and fostering collaboration, and it can involve a variety of methods, including qualitative interviews, surveys, focus groups, advisory boards, and participatory and codesign workshops. These various approaches to participant engagement may also conceptualize the role of participants quite differently; they may be sources of additional data, advisors, thought leaders, and even research partners.^3^ Such approaches also vary in the agency and power given to participants, not only over the research design but also over the assessment of outputs and the dissemination of findings. 

In other clinical research contexts, efforts to involve past or current participants in ways that go beyond their traditional role as passive subjects have been promoted with the promise of a wide range of positive outcomes,^4^ including clinical benefits such as better health outcomes for study participants or individuals in a targeted disease group), study benefits such as increased diversification of participants and better retention in long‐term studies), and translational benefits (such as better uptake of resulting interventions, increased public trust, and perceptions of research legitimacy).^5^ With respect to engagement as a general practice, many ethical rationales have also been offered. These include the potential for engagement to respect participants as persons by giving them greater agency, to minimize harm and empower marginalized groups by granting greater sovereignty over research results and their use, and to improve benefit profiles and considerations of justice by giving uptake^6^ to participants’ knowledge and lived experiences of an illness or disability.^7^

***Engagement in neural device research.*** Many of these motivations for engagement have been recognized in implanted neural device research, either explicitly or implicitly, and their general endorsement is evidenced by current engagement efforts sponsored by various entities, as we discuss below. Across these emerging initiatives, however, there are varying ideals and specificity about how engagement is defined, who is being engaged, and when engagement should happen.

For example, the FDA promotes engagement with “patient advisors,” defined as “individuals who have experience living with a disease or condition, and can serve in an advisory or consultative capacity to improve clinical study design and conduct, but who are not study/research participants themselves or caregivers of study/research participants.”^8^ The FDA recommends engaging patient advisors in a number of ways that can improve the design of clinical device trials, including defining meaningful end points for the treatment of specific diseases, advising on flexible options for follow‐up visits and data‐collection techniques, and improving informed consent documents for trials, among others. Engagement with patient advisors optimally occurs in the early planning phases of clinical research so that feedback may be incorporated with the goal of enhancing the design and conduct of clinical studies. However, FDA guidance explicitly advises against engagement with current study participants because of the potential for a conflict of interest, though the guidance does not describe the conflicts in detail.^9^

Some neurotechnology companies have established patient and community advisory boards with the intent of diversifying representation and accountability. For example, in April 2024, Motif Neurotech announced the formation of a community advisory board and the appointment of its first member, a recipient of deep‐brain stimulation and a survivor of depression.^10^ Other organizations, such as Neurotech Network, offer engagement services such as market analysis, engagement program design, community targeting, and other guidance on community engagement, defined as “engaging the end‐user community in the development of neurotech devices.”^11^ In 2024, the Implantable Brain‐Computer Interface Collaborative Community (iBCI‐CC) was formed, offering yet another model for engagement with various stakeholders—including people with lived experiences—in advancing guidance and best practices for the translation of brain‐computer interface (BCI) devices.^12^ But none of these efforts aim explicitly at engaging participants in the context of the studies in which they are currently enrolled, and it is far from clear how these efforts might be extended to do so.

The National Institutes of Health Brain Research Through Advancing Innovative Neurotechnologies (BRAIN) Initiative has identified participant engagement as a core area of neuroethics concern. In a recent request for applications (RFA) for grant proposals on ethical implications of neurotechnology research and advancement, “Research Participant Engagement, Special Populations, and Social Implications” is named as an area of neuroethics “central to the cutting‐edge research supported through the BRAIN Initiative.”^13^ The RFA explicitly cites the importance of participant perspectives on consent, risk, neuromodulation of brain function, and the distinction between invasive and noninvasive neurotechnologies, but no clear participant engagement methods or rationales are suggested, nor is it clear how gathering such perspectives would amount to engagement or achieve any of the benefits that engagement is often said to bring.

For over a decade, neuroethicists have relied on interviews with participants enrolled in implanted neural device studies during or after participation in research, commonly using qualitative research methods to analyze participant accounts of their lived experiences, values, and suggestions for device improvement.^14^ But these studies are not a sufficient form of participant engagement for several reasons.

First, they do not detail clear mechanisms by which participant perspectives will be incorporated into future research design. Although many end with calls for incorporating such perspectives into aspects of neural device research, it is unclear whether and how such recommendations will be implemented, as implementation likely depends on investigator uptake. To our knowledge, there exist very few studies aimed at the translation of the results of neuroethics qualitative studies into changes in neural device research.

Some ethics research team structures, such as embedded ethicists or clinician‐led ethics research, may facilitate greater uptake of participant perspectives into device research by fostering ongoing dialogue between researchers, ethicists, and participants.^15^ Yet such approaches remain relatively uncommon, and there is limited empirical evidence documenting their effectiveness in ensuring sustained participant influence on neurotechnology development. While embedded models may improve communication, much like qualitative research, they do not guarantee that participant feedback translates into tangible changes. This becomes especially apparent against the backdrop of ongoing power imbalances and various institutional hurdles. For example, participant feedback might reveal the need to modify the external hardware of a device to improve usability, but researchers have authority not to pursue those modifications, especially if they are judged to be too costly or would be time‐intensive modifications that need to be submitted to an institutional review board. Participants often have very little recourse to insist on them.

Second, the potential impact of qualitative studies and embedded ethics models depends on the interpretation of participant narratives and experiences by ethicists, who may be ignorant or “uncritical” of important technical and practical features of brain devices.^16^ These features may present real challenges to incorporating participant perspectives into device research.

Third, qualitative studies rarely explicitly aim at gathering participant input on critical features of device trials related to design, success metrics, or study processes. Instead, they are often more focused on questions related to risk‐benefit assessments, consent experiences, or post‐trial considerations. While the latter are certainly part of study design, they are often separated from the technical aspects of device or task design.

If these considerations are correct, then qualitative studies may risk imposing time and emotional burden on participants without a clear maximization of benefit to both current and future device users. In short, it seems that, while qualitative research provides useful information, it does not guarantee that this information is actually used, much less that the research will be altered in response to it.

***A way forward.*** There is, however, a growing recognition of the importance of soliciting user and potential end‐user input on various aspects of neural device design, especially regarding functionality, usability, and aesthetic appearance. It has been suggested that engaging potential end users of neural devices, at different phases of device development, may not only improve benefits for early users (including trial participants) but also yield better devices down the line. Incorporating user needs, values, and preferences also overlaps with commercial and design interests and aligns with concerns related to disability justice and the representation of disabled people in the development of neural devices.

Our recent qualitative study of participants in an early feasibility study for a visual cortical prosthesis provides an illustrative example of how current users’ perspectives could inform changes in study and device design that might have been missed in the study process. The narratives provided by current users enrolled in this feasibility study suggested a disconnect between the functionality of the prosthesis and participants’ assessment of the device's overall benefit. While the device functioned in accordance with its intended purpose to provide a form of artificial vision, participants did not consider it beneficial in daily life. This finding suggests the need for a more precise understanding of how users incorporate neural devices into daily life alongside other assistive technologies, as opposed to focusing solely on maximizing the quality of the artificial vision provided.^17^

The same study also found that all participants had concerns about the appearance of the external device components, with one person refusing to wear the device outside of their home. Similarly, a qualitative study of the assessment of BCIs from the perspective of people with paralysis found that aesthetic and cosmetic appearance factored heavily into patient preferences, even outweighing concerns about neurosurgical risks.^18^ This finding draws attention to the connection between aesthetic judgments and usability of neural devices and suggests the need for more emphasis on the importance of device appearance than may be given in current studies.

While engineers and ethicists have called for the implementation of a variety of user‐engagement practices in neural device research and design more directly, there is little consensus about best methods and collective practical guideance.^19^ For example, the Institute of Electrical and Electronic Engineers’ “Standards Roadmap: Neurotechnologies for Brain‐Machine Interfacing” discusses how the implementation of human factors engineering, usability engineering, and user‐centered design yields significant downstream benefits, including higher user satisfaction, better product adoption, and early insight about future products, features, and markets, but also acknowledges the financial burden of implementing these practices up front.^20^ At the same time, a qualitative study of BCI investigator perspectives on end‐user input found that, while investigators recognize the potential impact of end‐user values to BCI research, there is broad skepticism about selection bias and reliability of user‐centered design methods and varying views about how, when, and why to engage end users.^21^

The general momentum around user engagement and the broader emphasis on the value of lived experiences suggests a way of thinking more deeply about participant engagement, one that reveals how these practical motivations for end‐user engagement align with ethical commitments to justice. End‐user engagement appreciates the special epistemic status that participants have as early users of implanted neural devices, one that goes beyond unique perspectives, values, and opinions on consent, risks, and other traditional staples of neuroethics research. It appreciates that participants have special epistemological access to what it is like to use a device, what the optimal ways to use the device are, and how to derive the most benefit from their use. As we argue below, this lens should be expanded to motivate participant engagement as a matter of justice.

Before turning to this argument, it is worth highlighting that we regard engagement with implanted neural device participants as distinct from community engagement, patient engagement, and even end‐user engagement. We consider participant engagement as involving individuals who are, or have previously been, enrolled in a device trial and have therefore gained unique expertise, knowledge, and experience with a particular technology or therapy. Such participants may be involved in community engagement efforts or patient engagement efforts, but they need not be. But end users need not be current users, previous users, or participants in a study. Similarly, patient engagement may include participants in implanted neural device studies, since all participants are under clinical care for the duration of implanted neural device trials because of concerns about seizure, device malfunction, infection, or other risks. But this is not always the case, as engagement might aim to understand the experiences of blindness or loss of motor control and not necessarily the experiences of using a device meant for those experiences. End‐user engagement might target potential future users, which does not require their having been enrolled in a neural device study.

The distinction matters for a few reasons. It creates space to consider that the goals of community or patient engagement efforts might be different from those of participant engagement. It also creates space to recognize that, while each of these forms of engagement has valuable contributions to neural device research, they may require distinct engagement rationales and methods. And further, it makes space for considering that participants have a unique epistemological status, which is a point we will use to ground our argument.

As we will argue, participants are positioned to make crucial knowledge contributions in the development of implanted neural devices by virtue of their lived experiences of participating in these studies and their experiences living with the disabilities in which these devices seek to intervene. We propose that the epistemic lens reveals additional dimensions related to both the how and the why of participant engagement, which have been previously underexplored, and that returning to engagement through this lens may change both the motivations and proposed practices for engagement with participants. At the heart of this argument is the claim that conceiving of participants solely as passive research subjects rather than as epistemic partners is a form of participatory epistemic injustice.

## Contextualizing Epistemic Injustice

At its core, epistemic injustice concerns the ways in which structural and contextual power relations marginalize knowers from subjugated social groups and minimize the epistemic import of certain kinds of knowledge and modes of knowing, both in general and within specific epistemic domains, such as biomedicine and technology. For decades, feminist philosophers and philosophers of race, including Patricia Hill Collins, Marilyn Frye, Charles Mills, Patricia Williams, and Gayatri Spivak, among many others, have taken up the myriad ways in which dominant epistemological resources work to exclude certain knowers and deflate their credibility. These thinkers have emphasized how members of subjugated groups are excluded from participating in dominant knowledge‐making practices as well as the ways in which nondominant modes of knowing have been portrayed as illegitimate.

However, the term “epistemic injustice” was coined more recently, with Miranda Fricker's publication in 2007 of a book that offered the term as its title. Fricker identifies two key forms of epistemic injustice: testimonial injustice, which occurs when an individual's testimony is dismissed due to negative identity‐prejudicial stereotypes, and hermeneutical injustice, which occurs when an individual's or group's experience is obscured from collective understanding due to structural prejudices. Since the publication of Fricker's book, a wide body of philosophical literature has emerged specifying, with more nuance, various forms that epistemic injustice may take in specific domains of knowledge making.^22^ These distinctions are especially useful for understanding how epistemic injustice may appear in applied contexts such as neuroethics, neuroscience, and neurotechnology.

One form of epistemic injustice that is especially relevant to our concerns about engagement efforts in implanted neural device research is participatory epistemic injustice. Participatory epistemic injustice occurs through the exclusion of individuals and groups from shared epistemic activities due to forces of oppression, including those related to race, gender, and disability.^23^ Kidd and Carel have published several articles examining how ill and disabled persons may experience participatory injustice and how their perspectives may be marginalized within or entirely excluded from the production of knowledge in health care.^24^ They argue that, even when patient perspectives are sought out, if they are not formally taken up as epistemic contributions (for example, used to inform clinical guidelines, success criteria for new therapy, or clinical trial design), then patients may still experience participatory epistemic injustice. Kidd and Carel assert, “Despite initiatives such as patient and public involvement (PPI), co‐design methodologies, and patient‐centered care, by and large, such [patient] experiences still play little role in the design of clinical services, performance review, or production of clinical guidelines.”^25^

***Epistemic injustice and neural device research.*** As engagement efforts are launched in the implanted neural device sector, we worry that these initiatives may also fail to trigger action in similar ways. We are concerned that new engagement efforts may be performative and simply continue to gather participant perspectives without ensuring pathways for participant knowledge to impact important features of brain device research, such as device design, study design, study tasks, and success criteria. In what follows, we will explicate Kidd and Carel's argument and draw parallels to similar dynamics in participant engagement practices in neural device research.

Building on Christopher Hookway's account, Kidd and Carel begin with the premise that when we participate in collective epistemic practices, we hold both implicit and explicit expectations about the capacities of our epistemic peers. These include a belief that our epistemic peers have a sense of relevance—that is, they possess the capacity to determine which ideas are relevant to a specific epistemic project—and a belief that they have the capacity to provide information that can meaningfully contribute to an epistemic task—for example, the ability to interpret data or apply pertinent background knowledge on a given topic. Participatory injustice may occur when a group is prejudicially judged to lack the ability to discern which information is relevant or is unfairly judged to be incapable of providing relevant information in a shared epistemic project.

Kidd and Carel argue that patients are vulnerable to participatory epistemic injustice because they are judged to lack both a sense of relevance and a capacity to provide relevant information.^26^ Because patients often lack formal medical training and expertise, they may be judged to lack a sense of relevance for the epistemic practices of medicine and therefore also be viewed as unqualified to make meaningful epistemic contributions to projects like the development of neural devices. However, such judgments fail to acknowledge the ways in which patients’ lived experiences of illness offer insight into a sphere of relevance that may extend beyond what is apparent to clinicians, biomedical scientists, and other investigators, even if they are experts in a particular medical condition. This is because the patient's perspective on what is relevant, such as success parameters for a specific therapy, desired functional abilities, and desired pain reduction, arises from the lived context of illness, including how their condition impacts social, temporal, and spatial spheres of relation. As a result, the relevance and epistemic import of first‐person knowledge is often underappreciated in medicine. And, even if the value of patient knowledge is tacitly acknowledged, structural hierarchies in health care can prevent patient knowledge from having direct meaningful impact or from having such epistemic contributions formally recognized.

This distinction is particularly significant when considering the relevance of different domains of knowledge for the development of implanted neural device research. Early‐phase neural device research can be seen as a shared epistemic project that necessarily includes a wide range of epistemic contributions. In this research, teams may be comprised of clinical neurologists, neurosurgeons, psychiatrists, nonclinical neuroscientists, engineers, research coordinators, and stimulation technicians. Epistemic contributions include designing implanted device components, improving external device components and user devices, designing study protocols, gathering data, interpreting data, determining safety parameters, and establishing success criteria. Research team members make epistemic contributions, in part, on the basis of their expertise in specific domains.

Participants in implanted brain device trials are positioned to make crucial knowledge contributions in the development of neural devices, not only by virtue of their lived experiences with novel devices but also by virtue of their first‐person experiences of illness and disability. This knowledge should also be recognized as relevant to this epistemic project.

Kidd and Carel also argue that ill persons are vulnerable to participatory injustices because they are typically regarded as “the objects of, rather than participants in, the epistemic practices of medicine” such that they viewed as “sources of information” rather than “informants.”^27^ In the context of neural device development, participants may experience epistemic harm when they are not taken seriously as epistemic contributors and their contributions are not formally integrated into the research process in a way that ensures that they have decisional uptake, including the power to alter various aspects of device design and clinical trial procedures.

***Triggering action.*** What is key, according to Kidd and Carel, is not that patient perspectives are acknowledged, or even that they are collected, but that they are taken seriously as epistemic contributions, such that they “trigger action” in impactful ways. Existing methods of gathering patient perspectives, such as interviews and qualitative analysis, patient‐preferences studies, and even involving patient experts, may be insufficient. As they explain, “To assign someone a status of epistemic authority (‘patient expert’) is itself insufficient unless one also adjusts the wide structure of epistemic norms and practices to ‘build in’ those new authorities…. Expertise can be misconstrued and thought of individually; what is also needed are established roles for patient experts within the wider structures and practices.”^28^

Kidd and Carel imply that such epistemic injustice might be remedied if these perspectives were more readily incorporated into decisional apparatuses used across different domains of health care. Moreover, they stress that this requires structural changes to epistemic norms and practices, such as involving participants directly in the design of external brain device components. What it means for participants to be taken seriously as epistemic partners such that their contributions “trigger action” requires conceptual elaboration, especially in the specific context of implanted neural device research, which we do not take up here. Instead, we will expand Kidd and Carel's argument by clarifying the kinds of knowledge participants possess, how their knowledge is relevant to neural device development, and what might it mean, practically speaking, to grant them meaningful status as epistemic contributors.

## Contextualizing the Value of Participant Knowledge

Participant perspectives are often gathered through empirical ethics research with the goal of understanding values, preferences, and needs, rather than for their explicit value as epistemic contributions to the task of developing neural devices. It is not that patient preferences, values, and needs could not be part of an epistemic contribution but, rather, that they are typically elicited as part of ethics studies concerned with the moral conduct of neurotechnology research rather than as part of the neurotechnology research itself. We raise this point to expand the relevance of participant knowledge beyond the sphere of value theories to include potentially more impactful contributions to engineering and design.

Several features of neural device research amplify the relevance of participant knowledge, including the target populations of end users; the goals of eventual commercialization as a therapeutic or assistive technology; the capacity of neural devices to elicit new sensory experiences; the scarcity, or rarity, of participants; and the potential for these technologies to alter various existential categories, including how users relate to sense of self, others, and world.^29^ Developing functional, useful, usable, and marketable devices that may eventually benefit people with various disabilities and neurological conditions requires taking seriously participants as epistemic partners. This may involve reconceptualizing participants as key members of an epistemic community, rather than solely as passive research subjects. This shift in participant role requires taking seriously the relevance of the unique epistemic advantage afforded by the positionality that disabled trial participants occupy.

Resources from standpoint epistemology can help clarify this point. Standpoint epistemology begins with the premise that all knowers are situated, meaning that individuals have access to different kinds of knowledge based on their social positionality. Positionality includes basic categories like embodiment; first‐ versus third‐person perspective; geographical location; identity markers like race, gender, class, sexual orientation, and disability; professional titles; and membership in marginalized social groups that grant individuals insight into specific forms of oppression that impact those groups.

To explain the situated‐knowledge thesis, Briana Toole provides an example of two students who attend college in a large vertical city building and have classes on the top floor. The building has stairs, escalators, and elevators. Because Elsabeth is disabled and uses a wheelchair, she must use the elevator to get to class, whereas Janie can use stairs, escalator, or elevators. Elsabeth faces long elevator wait times and additional delays due to the lack of Americans with Disabilities Act‐compliant automatic door switches, which means that she must wait for another student to open the door when she is getting to class. When both students are asked to fill out an accessibility survey, Elsabeth details the features that impede her access to class. Janie reports that she doesn't know if there are accessibility issues, but “things seem fine to her.” Because of her situatedness as a disabled wheelchair user, Elsabeth has an epistemic advantage over Janie with respect to building accessibility.^30^

This scenario offers an analogy relevant to the development of neural devices: disabled participants who use neural devices are better positioned to know about features of device accessibility, usefulness, usability, functionality, and desirability than are nondisabled investigators. As disability studies scholar and bioethicist Rosemarie Garland Thomson stresses, disabled people have this kind of epistemic advantage precisely because they “misfit” with inaccessible built environments.^31^

Epistemic advantage and epistemic authority are useful concepts for understanding the different types of epistemic contributions that different stakeholders can make in the task of developing implanted neural devices. Following the situated‐knowledge thesis, epistemic advantage has to do with a person's ability to gain specialized knowledge by virtue of the subject position they occupy. While there is significant debate about the meaning of “epistemic advantage” and how it is achieved in the standpoint literature, for the purposes of our applied scenario, we highlight the relationship between the unique subject position participants occupy and the epistemic advantages it affords with respect to several domains of knowledge.

Epistemic authority is conferred by the social, political, and economic practices that render people to be seen as more reliable, more trustworthy, and more credible.^32^ While both participants and investigators are epistemically advantaged in different ways, only investigators—and specifically principal investigators—have been granted the epistemic authority to conduct neural device research, which includes making decisions about various aspects of the trial, such as the parameters of success and the design of the device. The relevance of principal investigators’ epistemic contributions is unquestioned by virtue of their subject‐matter expertise and professional titles. In contrast, the epistemic advantage of disabled participants is not formally acknowledged in the research team structure.

***Epistemic advantage and authority in neural device research.*** Given their positionality as individuals living with the disability or neurological condition in which the device is meant to intervene and as trial participants who have unique access to the sensory experience of a novel neural device, participants have an underappreciated epistemic advantage over investigators with respect to several domains of knowledge that are often underappreciated in neural device development. These are: 1) lived experience of disability; 2) lived experience of novel functionalities (sensory, motor) enabled by implanted neural devices; 3) knowledge about whether, how, and to what extent those functionalities are likely to be beneficial to disabled users; and 4) knowledge about what it's like to participate in novel device trials. This epistemic advantage is owed to their positionality as both individuals living with the disability or the neurological condition on which the device is meant to intervene and as trial participants who have unique access to the sensory experience of a novel neural device.

Participants’ first‐person knowledge about the disability or neurological condition in which the neural device aims to intervene includes both the embodied experience of that disability and the social experience of navigating inaccessible built and social environments, including structural barriers faced by disabled people related to inequalities in the health care system and broader social infrastructure. For example, blind participants have knowledge about what it is like to be blind—and, in some cases, to experience vision loss—in a world that is not designed for blind people. This affords them unique perspectives on the kinds of technological interventions that would be especially useful. Whereas a sighted person might assume that any restoration of visual function through an implanted visual prosthesis would be beneficial, a participant may have more nuanced and specific insight.

For example, during our qualitative study with participants in a trial for a visual cortical prosthesis, several participants reported using the device in conjunction with their white cane to navigate sidewalks and that having the device did not change how they used their white cane. Whereas sighted investigators, funders, and shareholders might imagine future versions of a visual cortical prosthesis replacing the white cane by restoring visual capacity, participants might imagine ways in which the neural device could be integrated with other assistive technologies to address specific problems they encounter in daily life.^33^

This point extends to other BCIs and neuroprostheses, including those that target speech and motor function. For example, participants with spinal cord injuries or those with tetraplegia may desire different functionalities from robotic arms than those that investigators find desirable, or they may see ways in which neuroprostheses could helpfully integrate with other assistive devices, such as wheelchairs and other mobility supports.

Disability theorists and activists have argued that disabled people are experts in the technologies they use. As bioethicist and amputee Ashley Shew stresses, “When we don't listen to people with actual experience, we often get accounts of disability *and* technology completely wrong. Disabled people are the ‘real experts’ …; when it comes to technology and disability. We use technologies, we also reject them, grapple with them, or repurpose them. The views on technology we get from listening to disabled people often look very different from those of people educated in the medical and helping professions.”^34^ However, the failure to center the epistemic contributions of participants, all of whom are disabled, in the early development of neural devices is unsurprising given the broader epistemic marginalization of disabled perspectives in the health care disciplines, including bioethics and neuroethics.^35^ Christine Wieseler argues that the exclusion of disabled perspectives from these disciplines goes beyond epistemic injustice and amounts to epistemic oppression because disabled people are persistently and structurally excluded from participation in epistemic communities that directly impact them.^36^

Recall that epistemic authority is conferred within existing systems and power structures that shape the conduct of biomedical research. When Kidd and Carel invoke the failure of patient‐perspectives studies to trigger action, they are referring to the fact that existing systems often fail to grant patients epistemic authority in domains of knowledge production that directly impact them, even when their epistemic advantage is acknowledged. To illustrate this point, Kidd and Carel provide a hypothetical case in which a group of rheumatic patients are invited to sit as observers on a committee reviewing a physiotherapy program available to them. Although they have meaningful insight into how the success criteria for the program should be determined, their status as invited observers means they have no power to vote and cannot directly impact the committee's decisions.^37^ In their example, the patient's epistemic advantage is recognized insofar as they are invited to sit on the committee and share their views, but they are not given voting status and thus lack decisional agency regarding the epistemic output of the committee—recommendations for the improvement of the physiotherapy program. Instead, the patients are left to influence voting committee members indirectly.

There are several factors that might minimize the indirect influence of the patient group on the committee—preexisting social dynamics on the committee, personality traits such as shyness or timidity among the members of the patient group, bias against disabled people, and bias in favor of medical models of disability, to name a few. Without voting status, patients’ influence depends on committee members’ receptivity to patient perspectives and willingness to advocate for the patient group. Due to the structure of the committee, epistemic injustice persists despite acknowledgement of the value of patient knowledge.

This scenario demonstrates the limitations of simply recognizing the relevance of participant knowledge without modifying structures to facilitate the uptake of the participants’ epistemic contributions. Since belonging to a voting committee is not the only means of making an epistemic contribution, we prefer the terms “decisional agency” and “decisional status” over “voting status” to signify a more agential and impactful mode of epistemic contribution. Acknowledgement of unique epistemic advantage alone does not remedy epistemic injustice; structural changes are required.

We can use the lens of Kidd and Carel's example to reconsider ethics studies that solicit the perspectives of brain device trial participants. As discussed earlier, these studies elicit participant perspectives on a variety of issues but fall short of providing formal pathways by which this knowledge is reincorporated into brain device research. Engagement efforts that only recognize the value of participant knowledge but do not attempt structural changes risk being performative. Given these points, we argue that failure to grant decisional status to disabled participants’ unique and expert knowledge of neural technologies with respect to the design and improvement of neural devices amounts to epistemic injustice. Engagement efforts may offer some opportunities to remedy this widespread dynamic, but only if structural changes to the research processes are attempted.

Some may argue that participants are already viewed as epistemic contributors rather than as passive subjects because the nature of implanted neural device research requires that the participants continuously provide feedback to investigators. However, while existing trial structures place high demands on participants, they do not necessarily entail that participants’ contributions are taken seriously as epistemic partners. This is because these early phase device trials retain a unilateral and asymmetrical decisional structure. Instead, recognizing participants as epistemic partners requires structural changes that grant participants decisional agency on the grounds of their epistemic contributions to the research.

This would involve, among other things, moving away from an extractive model of gathering feedback in which participants are understood as sources of information toward a more active form of epistemic collaboration—in short, treating participants as research partners rather than as research subjects. This might include, for example, soliciting participant or end‐user feedback on device design before a prototype is locked down for multiyear trials. We see participant engagement as one potential avenue for accomplishing this but maintain that current feedback mechanisms are inadequate.

## Implications for Engagement

The recent proliferation of engagement efforts in implanted neural device research offers important opportunities to remedy participatory epistemic injustice for participants. While more targeted research about best engagement practices remains imperative, we hope to have elucidated how the epistemic dimensions of this research intersect with ethical imperatives for engagement with neural device research participants. Centering epistemic injustice as a motivation for engagement has implications, not only for why device developers should pursue engagement but also for how to go about it, including which engagement methods should be used and how they should be implemented. More research is needed in these areas.

Conceptualizing participants as crucial epistemic partners—that is, as key members of the research team—may also have practical implications for various other neuroethical topics. If those of us who are involved in neurotech research change how we consider participants as epistemic peers in the project of implanted neural device development because participants possess expertise in the aforementioned ways, this may change how we think about other elements of trial design, including recruitment, consent, post‐trial obligations, compensation, the determination of meaningful clinical outcomes, and other related ethical concerns. This change may also entail acknowledging participants’ contributions as labor and providing the corollary material supports afforded to others who labor in these tasks.

## Acknowledgments

This article is part of a series, Neuroscience and Society, whose development by The Hastings Center is funded by the Dana Foundation.

The authors’ work was supported by National Institutes of Health grant RF1MH121373 and a grant from the Dana Foundation.
